# Collectanea Medica: Consisting of Anecdotes, Facts, Extracts, Illustrations, &c.

**Published:** 1825-02

**Authors:** 


### COLLECTANEA MEDICA:

CONSISTING OF

### ANECDOTES, FACTS, EXTRACTS, ILLUSTRATIONS, &c.

*Relating to the History or the Art of Medicine, and the Collateral Sciences.*

Floriferis, ut apes, in saltibus omnia libant,

Omnia nos, itidem, depascimur aurea dicta.

Art. I.—*Facts towards the Chemical History of Mercury.*—(From the Journal of Science, No. xxxvi.)

### § I. *Of the Oxides of Mercury.*

Some valuable information respecting the oxides and sulphurets of mercury has been published by M. Guibourt, in the Journal de Pharmacie, of the year 1816; but we cannot agree with his conclusion as to the impossibility of obtaining an insulated protoxide, and an insulated protosulphuret, of that metal: at least, if we understand him rightly, he considers those compounds as mixtures of metallic mercury with the peroxide and bisulphuret respectively; and this, because they are so easily resolved by heat, and even by trituration, into running mercury and peroxide, and running mercury and bisulphuret. But we do not reject iodide of nitrogen, or fulminating silver or mercury, from the list of chemical compounds, because slight mechanical means suffice to decompose them.

Calomel boiled in excess of solution of soda, or treated with excess of ammonia, yields a black powder, which, if carefully dried out of the contact of air, affords, on decomposition, about ninety-six per cent. of mercury, and four of oxygen; or 200 of mercury and *eight* of oxygen; while the red oxide of mercury, a crystalline, permanent, and definite compound, beyond all doubt, affords 200 of mercury and *sixteen* of oxygen. There is, therefore, every reason for adopting the former as a true combination; for we find that it is entirely soluble in, and forms distinct salts with, acids, and that it contains just half the proportion of oxygen existing in the peroxide. It is nevertheless true, that by a gentle heat, by trituration, or by exposure to light, this protoxide throws off a portion of its mercury, whilst the remainder of the metal passes into the state of peroxide.

### § II. *Of the Sulphurets of Mercury.*

In respect to the sulphurets of mercury, M. Guibourt is of opinion that the protosulphuret is to be considered as a mixture *(un mélange)* of cinnabar and metallic mercury, because it is as easily decomposed as the protoxide: nevertheless, he shows that it consists of 200 of mercury and *sixteen* of sulphur, and that the bisulphuret, or cinnabar, is composed of 200 mercury and *thirty-two* sulphur. There seems, therefore, no reason whatever for assuming the protosulphuret to be an indefinite mixture; and, if it be considered, as some would have it, a *compound* of cinnabar and mercury, what is that but a protosulphuret, as proved by its analysis? Respecting the mode of obtaining the protosulphuret, there may, however, be some difference of opinion. It appears to us that the only method by which such a definite compound can be procured, consists in passing sulphuretted hydrogen gas into a very dilute solution of the protonitrate of mercury, or into water through which calomel is diffused: in either case, a perfectly black precipitate is formed, which, when collected upon a filter, and very carefully dried, exhibits no globules of mercury, and which, when analysed, affords results consistent with one proportional of mercury and *one* of sulphur. Like the protoxide, however, it is singularly easy of decomposition. Trituration effects this; so does exposure to the sun's rays; so does a heat of 300°; and, when heated in a glass tube in the flame of a spirit-lamp, mercury distils over, and bisulphuret is sublimed.

The pharmaceutical preparation, called Æthiop's mineral, is generally stated to be a mixture of sulphur and black sulphuret of mercury; indeed, it is called, in the latest edition of the London Pharmacopœia, "Hydrargyri Sulphuretum Nigrum." It has long had a place in the materia medica, though a very useless and inert compound. It is a black powder, prepared by triturating together equal weights of mercury and sulphur, until globules are no longer visible. When properly made, it may be rubbed upon gold, without leaving any mercurial stain upon that metal: hence it appears that the mercury is in chemical combination with the sulphur; a circumstance, indeed, sufficiently evident from the great rise of temperature that ensues when the materials are subjected, in large quantities, to powerful trituration; they then smoke and agglutinate. Fourcroy and several other chemists have regarded this compound as consisting of sulphur and black oxide of mercury; but Prout and others have amply demonstrated the fallacy of such an opinion, without, however, showing the real nature of the substance. It has lately been usual to describe it as a mixture of sulphur and black, or protosulphuret of mercury; and it is stated, in several pharmaceutical works of authority,* to be "insoluble in nitric acid, but totally dissolved by a solution of pure potassa." An inquiry into the truth of this assertion, so contrary to what one would expect *à priori*, has, I think, led to a satisfactory explanation of the nature of Ethiops mineral.

A portion of well-prepared Ethiops mineral was boiled in solution of potassa, until no further action took place; the liquor was then decanted off, and fresh portions of the alkaline solution added; the boiling being repeated as before, until no further action ensued. There remained a black powder, not acted upon by strong solution of potassa, nor by nitric nor muriatic acid. It was collected upon a filter, thoroughly edulcorated, and dried in a very moderate heat, not any time exceeding

* DUNCAN'S *Edinburgh New Dispensatory;* THOMSON'S *London Dispensatory;* PARIS, *Pharmacologia.*

212°; it was then of a very dark violet hue: when heated in a glass tube, it sublimed without any evolution of mercury, and became perfect cinnabar. It appears, therefore, that Ethiops mineral is far from being soluble without decomposition in alkaline menstrua: on the contrary, solution of potassa merely abstracts its excess of sulphur, furnishing a liquid sulphuretted hydrosulphuret of potassa, from which the acids throw down sulphur, but which affords no traces of mercury. When, however, moist bisulphuret of mercury, as it is left by the alkaline solution, is boiled in a strong solution of hydrosulphuret of potassa, a minute quantity of it appears to be taken up.

There are some discrepancies in the chemical history of the bisulphuret of mercury or cinnabar, as given in the above-quoted works, which the following facts may, perhaps, tend to clear up.

Cinnabar is not altered (except slightly as to colour, which is somewhat brightened whilst it is moist,) by long-continued boiling in nitric acid; nor is it affected by muriatic acid under similar circumstances. Nitro-muriatic acid instantly attacks it even in the cold, and by boiling converts it into a persulphate of mercury. If the sulphuric acid be precipitated by muriate of baryta, it is found that eighty parts are obtained from 232 of cinnabar, indicating the existence of two proportionals of sulphur to one of mercury, as above stated.* Sulphuric acid is partially decomposed when boiled upon cinnabar; sulphurous acid gas is given out, and, upon boiling down to dryness, a white sulphate of mercury is ultimately obtained. The non-action of nitric acid upon cinnabar, considering the facility with which it oxidizes and acidifies its elements, is remarkable; and it deserves notice, that when the black protosulphuret of mercury (obtained by precipitation from the protonitrate by sulphuretted hydrogen,) is boiled with that acid, that it is speedily decomposed and entirely converted into nitrate of mercury, and not a particle of cinnabar is separated, which probably would be the case if M. Guibourt's view of its composition were correct.

### § III. *Of the Chlorides of Mercury.*

It is now universally admitted, that calomel and corrosive sublimate are chlorides of mercury; that calomel, or the protochloride, consists of one proportional of mercury = 200, and one of chlorine = 36; and that corrosive sublimate, or the perchloride, consists of one proportional of mercury = 200, and two of chlorine = 72: the equivalent or representative number, therefore, of the former, is 236, and of the latter 272.

In some inquiries connected with the preparation of calomel upon the large scale, conducted in the laboratories at Apothecaries' Hall, Mr.

---

* In this analysis of cinnabar, it was found that 232 grains of cinnabar, after having been acidified by nitro-muriatic acid, required exactly 248 grains of crystallised muriate of baryta for the precipitation of 236 grains of sulphate of baryta. The correctness, therefore, of Mr. Phillips's equivalent for crystallised muriate of baryta may be considered as established,—viz. that it consists of one proportional of dry muriate (chloride of barium) = 106 + 2 proportionals of water $(9 \times 2 = 18) = 124$.

Hennell has discovered several curious and important facts respecting the chlorides of mercury, more especially in relation to the triple compounds formed by corrosive sublimate with other chlorides. He has ascertained that certain chlorides, which appear to have no action upon calomel at common temperatures, decompose it at a boiling heat, to a greater or less extent, and resolve it into corrosive sublimate and metallic mercury.*

This action he has particularly investigated in respect to common salt and muriate of ammonia, those being the substances usually employed for the purpose of washing calomel, under the idea of freeing it from corrosive sublimate; an effect which they fulfil when employed cold and in dilute solution only. But, when perfectly pure calomel is boiled for a few minutes in a solution of muriate of ammonia or of common salt, and a portion of the liquor filtered off and tested, a portion of sublimate is always found in it; and, on boiling for a long time, the whole of the calomel is decomposed, and compounds of sal ammoniac and corrosive sublimate, and of common salt and corrosive sublimate, are obtained; an equivalent portion of metallic mercury being at the same time separated.

These facts are peculiarly important in relation to the preparation of calomel, inasmuch as the Pharmacopœia directs the use of a hot solution of muriate of ammonia, with the intention of freeing it from any accidental admixture of corrosive sublimate; and Dr. Henry, in describing the methods of ascertaining the purity of calomel, directs it to be boiled in solution of muriate of ammonia. "When carbonate of potassa," he observes, "is added to the filtered solution, no precipitation will ensue, if the calomel be pure."† Several other chemists of eminence have given this is as a criterion by which to recognise the presence of corrosive sublimate in calomel; whereas it appears, from Mr. Hennell's experiments, that the protochloride of mercury is in such cases decomposed, and that perchloride is formed.

The compounds of corrosive sublimate with other chlorides, though

* Since writing the above, the following note has been received from Mr. Hennell:—

"I had repeatedly noticed a bluish tinge which calomel acquires when washed in a boiling solution of muriate of ammonia, as directed by the London Pharmacopœia, to remove corrosive sublimate. To ascertain the cause, I boiled 100 grains of pure calomel in a solution of muriate of ammonia, containing 100 grains of the salt. The change of colour in a few minutes was very evident. The solution, when tested, contained corrosive sublimate. The boiling was continued with four other portions of muriate of ammonia, 100 grains each; when the calomel was entirely decomposed, forty grains of mercury remained. Sixty grains of white precipitate were obtained from the solutions by carbonate of soda. There was no decomposition of the sal ammoniac. With common salt I obtained the same results,—mercury remaining, and white precipitate being thrown down from the solutions, by liquid ammonia. Common salt is not so active in producing these changes; as *ten* portions of 100 grains each were used, before the decompositon of 100 grains of calomel was complete. Muriate of potass and the earthy muriates have, I have every reason to believe, the same power; but I did not push the experiments as in the case of soda and ammonia."

† *Elements of Experimental Chemistry*, 9th edition, p. 588.

noticed by many authors, and more particularly by Dr. Davy, in his paper printed in the Philosophical Transactions for the year 1822, have not hitherto been submitted to analysis, nor examined with much precision. Mr. Hennell has already obtained some interesting results upon this subject, of which we hope to be able to give an account on a future occasion. In examining the mutual action of muriate of ammonia and corrosive sublimate, his attention was naturally directed to the substance usually called ***white precipitate***, the "Hydrargyrum præcipitatum album" of the Pharmacopœia: his experiments appear to me to leave little doubt that it consists of one proportional of peroxide of mercury, and one of muriate of ammonia.*

---

ART. II.—***Experiments made upon six decapitated Robbers;*** by Professor BARTELS.—(From the Edinburgh Philosophical Journal, No. xxiii.)

ON the 14th of October, 1811, six highway robbers were beheaded near Marburg; one of them was sixty years of age, the other five from twenty to thirty. At the instant when the head of the first fell, the trunk got up again, as if the individual had been about to rise upon his feet; while the bodies of the others fell down flat at the very moment. When, a little after, the heads were thrown at the foot of the scaffold, we saw all the muscles of the face of the last-executed completely relax, while those of the old man presented a general contraction, which lasted for a considerable time. These opposite effects took place without the

---

* Having inferred from previous experiments, that the "white precipitate" was a compound of one proportional of peroxide of mercury and one of muriate of ammonia, Mr. Hennell verified his opinion as follows:—A solution of one proportional of corrosive sublimate ($= 272$) was mixed with a quantity of solution of ammonia, containing two proportionals ($17 \times 2 = 34$) of that alkali; a neutral mixture resulted, white precipitate was formed, and one proportional of muriate of ammonia, (ammonia $17 +$ mnriatic acid $37 = 54$ of muriate of ammonia,) was found in solution. In this case, the two proportionals of chlorine in the sublimate ($36 \times 2 = 72$) were converted, at the expense of two proportionals of water, into two of muriatic acid, which, uniting with the ammonia, formed two of muriate of ammonia. The two proportionals of the oxygen from the water (equivalent to the two of hydrogen transferred to the chlorine,) united to the one proportional of mercury in the sublimate, to form one of peroxide of mercury; which fell, in combination with one of muriate of ammonia, to constitute white precipitate; while the other proportional of muriate remained, as above stated, in solution. The equivalent number, therefore, of white precipitate, is 270, and it consists of

| | | |
|---|---|---|
| One proportional of peroxide of mercury | = 216 | 80 |
| One ———— muriate of ammonia | = 54 | 20 |
| | 270 | 100 |

Having thus synthetically established the composition of white precipitate, the following analytical experiment was made upon it:—270 grains were dissolved in hydrocyanic acid, and sulphuretted hydrogen was passed through the solution till it occasioned no further change; the precipitate was then collected, washed, and dried; it weighed very nearly 232 grains, being the equivalent of bisulphuret of mercury. The filtered liquor, on evaporation to dryness, left fifty-four grains, or one proportional of muriate of ammonia.

occurrence of any difference in the mode of decapitation, to which they could be attributed. With respect to this, it will not be useless to remark, that there remained at least two vertebræ attached to each of the heads. It was observed that, at the moment of decapitation, the muscles of the face of the greater number of the heads contracted in a convulsive manner.

As the head of the first decapitated had not been brought in with the rest, no other observations were made with regard to it.

The second, which fell ten minutes after it, was observed without loss of time. It was tried at first to excite a contraction of the iris, by pricking that organ ; but no apparent motion was obtained. The same operation having been made upon the iris of the third head, the pupil dilated a little, and again quickly contracted ; while, at the same time, the pupil of the other eye (which had not been pricked) contracted, and again immediately dilated: an effect which Professor Trenderoth, as well as Messrs. Bunger and Herold, who were also present, saw in the most evident manner.

Some minutes after decapitation, the bodies were opened: the heart contracted and dilated alternately with much force, in such a manner as to produce regular pulsations. At the end of ten minutes, these motions had, it is true, abated a little; but they were always incessant, and the alternate contraction and dilatation preserved their regularity. Five minutes later, these motions had become unequal and very weak; they revived, however, when the heart was irritated by pinching it. A mechanical irritation, made upon a branch of the great sympathetic, accelerated a little the motion of the heart, but only for a minute at most: the motion itself, however, continued for a long time, only decreasing in intensity. A puncture made in the transverse muscle of the abdomen of the same body occasioned strong convulsions, especially in the lower extremities; and yet the nerves had not been immediately irritated. A mechanical irritation made at the lower part of the spinal marrow, caused violent contractions in the muscles of the trunk, as well as in those of the neck, particularly those of the upper part, at the place of the section, (which had already been frequently remarked.)

On irritating the upper part of the spinal marrow of another head, convulsive motions were produced in the muscles of the face, and there resulted a movement of the tongue and surrounding muscles.

In the third body, a motion was remarked in the lower part of the trachea which remained attached to the trunk: this motion was accompanied with a sort of hissing,—an effect caused, without doubt, by the convulsive contractions of the muscles which had been cut. Similar motions took place in all the others.

The head of the last decapitated was transported to the theatre, which, on account of the distance, occasioned the loss of an hour. Here, our first care was to try the duration of the galvanic irritation upon the different muscles of the head. The elevator muscle of the upper eyelid, and the superior oblique muscle, no longer contracted; but the frontal muscles, the orbicularis palpebrarum, masseter, digastric, &c. still continued to contract. The contractions ceased first in the masseter muscle; they were prolonged in the buccinator. Two hours after execution, it had entirely ceased in all the muscles, and it could not be excited on moistening them anew.—In another head, cut off twenty minutes at least before the preceding, the galvanic irritation caused the depressor commissure labiarum, the orbicularis palpebrarum, and masseter, to contract; this latter always much longer than the others. Two hours and three-quarters after decapitation, the muscles of this head appeared to have lost all irritability.

Before concluding our experiments upon the head of the last decapitated, we exposed the pectoralis major and minor of a body which was brought in. The large pectoral muscle alone contracted under the influence of the galvanic fluid, the muscles of the abdomen no longer contracted. Contraction took place only in the right triceps muscle and in the sartorius; they ceased always, in the latter, half an hour sooner than in the other. Irritation applied to the transverse muscle of this body no longer produced contraction, which we attributed to the circumstance that the body had been opened at the place of execution, after the first experiment.

In another body, which had been opened at the same time, the application of galvanism also produced some motions, as well as a feeble contraction, which was not renewed: mechanical irritation produced none.

An hour and a half after execution, the natural motion of the heart had ceased in the bodies already carried to the theatre. We were still, however, in hopes to produce contraction by means of irritation: not being able to get at the heart of the body which had been first opened, we proceeded to that of a body which had been newly opened. This last had also retained its heat, principally in the internal parts: the heart still retained a little blood, of a deep colour, in the left ventricle, which was partly fluid and partly coagulated; but we could not, either mechanically or by means of galvanism, excite any contraction of the muscular fibres of the heart.—(*Schriften der Gesell der Gesammt Naturwiss zu Marburg*, vol. i. 1823.)

